# Chicoric acid ameliorate inflammation and oxidative stress in Lipopolysaccharide and d‐galactosamine induced acute liver injury

**DOI:** 10.1111/jcmm.14935

**Published:** 2020-01-27

**Authors:** Zheng Li, Haihua Feng, Lu Han, Lu Ding, Bingyu Shen, Ye Tian, Lilei Zhao, Meiyu Jin, Qi Wang, Haiyan Qin, Jiaqi Cheng, Guowen Liu

**Affiliations:** ^1^ Key Laboratory of Zoonosis Ministry of Education College of Veterinary Medicine Jilin University Changchun China

**Keywords:** acute liver injury, AMPK, autophagy, CA, d‐GalN, LPS, MAPK, NF‐κB, Nrf2

## Abstract

Chicoric acid is polyphenol of natural plant and has a variety of bioactivity. Caused by various kinds of stimulating factors, acute liver injury has high fatality rate. The effect of chicoric acid in acute liver injury induced by Lipopolysaccharide (LPS) and d‐galactosamine (d‐GalN) was investigated in this study. The results showed that CA decreased the aspartate aminotransferase (AST) and alanine aminotransferase (ALT) in serum and reduced the mortality induced by LPS/d‐GalN. CA can restrain mitogen‐activated protein kinases (MAPKs) and nuclear factor‐kappa B (NF‐κB) to alleviate inflammation. Meanwhile, the results indicated CA can active nuclear factor‐erythroid 2‐related factor 2 (Nrf2) pathway with increasing the level of AMP‐activated protein kinase (AMPK). And with the treatment of CA, protein levels of autophagy genes were obvious improved. The results of experiments indicate that CA has protective effect in liver injury, and the activation of AMPK and autophagy may make sense.

## 1 INTRODUCTION

Acute liver injury, which is also named acute liver failure (ALF), was caused by extrahepatic and intrahepatic factors (such as alcohol, drug and surgery) with plenty of complication without available drug.^1^ In general, the progress of ALF is urgent and makes liver irreversible injury. For specific causes, ALF may have particular treatment, but the majority of patients have vague cause, which leads to only providing supporting therapy for them. And the most common treatment is liver transplantation result from irreversible cell injury. Recently, AFL is not only common in human but also has been taken seriously in animals. LPS, one of elements of outer membrane in Gram‐negative bacteria, can destroy the cell homoeostasis. To exacerbate ALF, d‐GalN can amplify toxicity of LPS. This model is similar with liver failure in clinical. Oxidative stress and inflammation speed up the occurrence and progress of various liver diseases. The connection of oxidative stress and inflammation draws great attention in progress of illness. It has been reported that reactive oxygen effects on the signal transduction pathways to induce liver injury caused by oxidant.^2^ NF‐κB family participates in inflammation and has crosstalk with other pathway, such as p38 (MAPKs family) and Glycogen synthase kinase 3β (GSK‐3β).^3^ MAPKs, which include extracellular signal‐regulated kinases 1 and 2 (ERK1/2), c‐Jun NH2‐terminal kinases 1, 2 and 3 (JNK1/2/3) and p38α/β/δ/γ, participate in numerous metabolic processes in liver. The activated JNK mediates liver injury though TNF and oxidative stress, which lead to hepatocytosis.^4^ Phosphorylated ERK is significant increased in models of ALF_,_ 5 and the disordered P‐ERK can aggravate liver injury. It has been proven that p38 is associated with inflammation^6^, ^7^ and oxidative stress. Besides, inflammasome complexes NLRP3 can activate IL‐1β, which is essential in congenital and adaptive immunity. Previous study has proven that the activated NLRP3 can aggravate the liver injury by intense inflammation.^8^

AMPK makes sense of maintaining energy balance in cell. Present study reports that AMPK is activated to respond the oxidative stress in cancer cells and neurons.^9^, ^10^ Meanwhile, it is reported that AMPK can increase the nuclear accumulation of Nrf2 though activating phosphorylation of GSK3β.^11^ GSK3β is a key enzyme in glucose metabolism, and the participation of it in cell regulation has been proven. Nrf2 is a transcription factor which can response oxidative stress. After transferring into nucleus, Nrf2 can coordinate a large amount of gene expression which responds to oxidative stress. Nrf2‐dependent genes and proteins, such as Heme oxygenase‐1 (HO‐1) and NADPH quinone oxidoreductase 1 (NQO1), are increased whereas Nrf2 transfers to nucleus and binds with Cis‐acting androgen responsive elements (AREs) to activate the expression of phase II detoxifying enzymes.^12^ HO‐1 shows abundant effects in responding to lots of stimulant. For GCL is an essential enzyme in assembling GSH, high level GCL reflects ability in confrontation of oxidative stress. Multiple factors (such as lack of nutrition and tissue injury) can induce autophagy, which can adjust the progress of disease. And autophagy can eliminate the damaged organelle and protein to protect cell form injury and death.^13^, ^14^ Therefore, autophagy suggests that it can have positive effects in diseases (such as cancer and cardiopathy). In recent years, researches of autophagy are significant in liver cell, and it is hotspots in liver injury. Present research was shown that the autophagy associated proteins was adjusted by redox balance regulation, which is relied on Nrf2 pathway.^14^

Natural product is a great source of drug, and they have potential in drug screening. Chicoric acid, one of Polyphenol, is extracted from Cichorium intybus L and other plants. Present experiments have been proven that chicoric acid can relieve the inflammation and oxidative stress induced by plenty of irritant, such as LPS and other irritant.^15^, ^16^ In our experiment, we detect the effect of CA in ALF.

## 2 MATERIALS AND METHODS

### 2.1 Materials

Chicoric acid (Cat #6537‐80‐0) was purchased from Chengdu Pufei De Biotech co., Ltd (Chengdu, China). We bought d‐galactosamine hydrochloride (Cat #1772‐03‐8) from Aladdin Industrial Corporation (Shanghai, China). Dimethylsulphoxide (DMSO) (Cat #D5879) and LPS (Escherichia coli lipopolysaccharide, 055:B5 Cat #L6529) were acquired by from Sigma‐Aldrich. We got ALT (Cat #C009‐2‐1), ALT (Cat #C009‐2‐1), glutathione (Cat #A006‐2‐1) detection kits and myeloperoxidase (Cat #A044‐1‐1) detection kits from the Jiancheng Bioengineering Institute of Nanjing. We brought glutathione (Cat BC1170) detection kits form Beijing Solarbio Science & Technology Co., Ltd. Anti‐HO‐1 (Cat #ab68477, RRID:AB_11156457）, GCLC (Cat #ab126704, RRID:AB_11127439), GCLM (Cat #ab126704, RRID:AB_11127439), NQO1 (Cat #ab80588, RRID:AB_1603750) and anti‐Nrf2 (Cat# ab62352, RRID:AB_944418) monoclonal antibodies were obtained from Abcam. We gained MAPK family antibody sampler kit (Cat #9926, RRID:AB_330797), phospho‐MAPK family antibody sampler kit (Cat #9926, RRID:AB_330797), autophagy antibody sampler kit (Cat #4445, RRID:AB_1196643), phosphorylation‐AMPKα (Cat #2535, RRID:AB_331250), AMPKα (Cat #5831, RRID:AB_10622186), phosphorylation‐AMPKβ (Cat #4186, RRID:AB_2169737), AMPKβ (Cat# 12 063, RRID:AB_2797812), IκBα (Cat #4812, RRID:AB_10694416), phosphorylation‐IκBα (Cat #9246, RRID:AB_2267145), NF‐κB (Cat #8242, RRID:AB_10859369) and phosphorylation‐NF‐κB (Cat #3033, RRID:AB_331284) from Cell signal technology (Boston, MA, USA). Anti‐Lamin B (Cat #12987‐1‐AP, RRID:AB_2136290), anti‐β‐actin (Cat #60008‐1‐Ig, RRID:AB_2289225) monoclonal antibodies were purchased from Proteintech Group Inc. HRP‐conjugated goat anti‐rabbit (Cat #BA1054, RRID:AB_2734136) and goat antimouse (Cat #BA1050‐1, RRID:AB_10892412) antibodies were provided by Boster Biological Technology. All other chemicals were of reagent grade.

### 2.2 Animals

Male C57BL/6 mice (6‐8 weeks old, weighting approximately 18‐22 g each) were purchased from the Liaoning Changsheng Biotechnology. With enough food and water, mice were kept in a comfortable environment. (24 ± 2°C, 55 ± 10% humidity).

### 2.3 Method

The mice were given LPS (30 μg/kg) and d‐GalN (600 mg/kg) with 400 μL saline solution (intraperitoneal injection) for construction of ALF. We have evaluated the effect of Chicoric acid (50 mg/kg) though giving the Chicoric acid to mice, which were in therapeutic group, before giving LPS and d‐GalN (1 hour). We collected the blood of mice 3h and 6h later (after injection of LPS and d‐GalN) though retro‐orbital venous plexus. Meanwhile, we removed the liver for histopathological examination and protein detection. In control group, mice were injected saline solution. Treatment of CA (50 mg/kg) was used to mice for negative control. We used 5% DMSO to dissolve CA. In this study, the experiments of mice were approved by the Animal Welfare and Research Ethics Committee at Jilin University and the experiment operation conformed experimental standards.

### 2.4 Serum ALT, AST and ROS level

The blood was pleased in 37°C for 2 hours, and then, it was centrifuged for 10min with 1000*g* (4°C). Under the instruction, we detected the ALT, AST and ROS in serum with kit.

### 2.5 Biochemical analyses

The removed liver tissue was operated with instruction. Then, we detected absorbance with spectrophotometer at special wavelength. And the activity of MPO and GSH was measured though the formula.

### 2.6 Histopathological evaluation

Part of liver tissue in each group was isolated and fixed for 48 hours with normal 10% neutral buffered formalin. After treated by ethanol and xylene, the liver was encapsulated in wax. We used slice to evaluate the injury after staining sections by haematoxylin and eosin.

### 2.7 Cell culture

The human hepatocellular carcinoma cell line HepG2 was obtained by China Cell Line Bank. HepG2 was cultured by DMEM with 12% FCS 1% penicillin‐streptomycin (5% CO_2,_ 37°C).

### 2.8 MTT assay

HepG2 (4 × 10^5^ cells/mL) was plated onto 96‐well plate. After cultured 24 hours, we used different concentrations of CA (0‐800 μm) to treat cell. Cultured for 24 hours, 20 μL MTT was added into the plate for 4 hours. 150 μL DMSO was added into each plate without other liquid. The optical density was measured by microplate reader (570 nm).

### 2.9 Western blot

The collected cell and liver tissue was treated by cell lysis solution. And then, we used BCA protein assay kit (Thermo, America) to detect the concentration. With the help of 10% SDS‐polyacrylamide gel, target substance was obtained and it was transferred to polyvinylidene fluoride membrane. After blocked, incubated by particular primary antibody and secondary antibody, we analysed the bolt with Image‐Pro Plus, and we detected the signal with the assistance of ECL (Dalian Meilun Biotechnology Co., Ltd).

### 2.10 Statistical analysis

Three independent data of experiment were collected and applied for one‐way ANOVA by Statistical Product and Service Solutions. *P* < .05 or *P* < .01 makes sense in statistics. The data were presented as mean ± SD.

## 3 RESULTS

### 3.1 CA decreases serum AST, ALT and ROS

**Figure 1:**
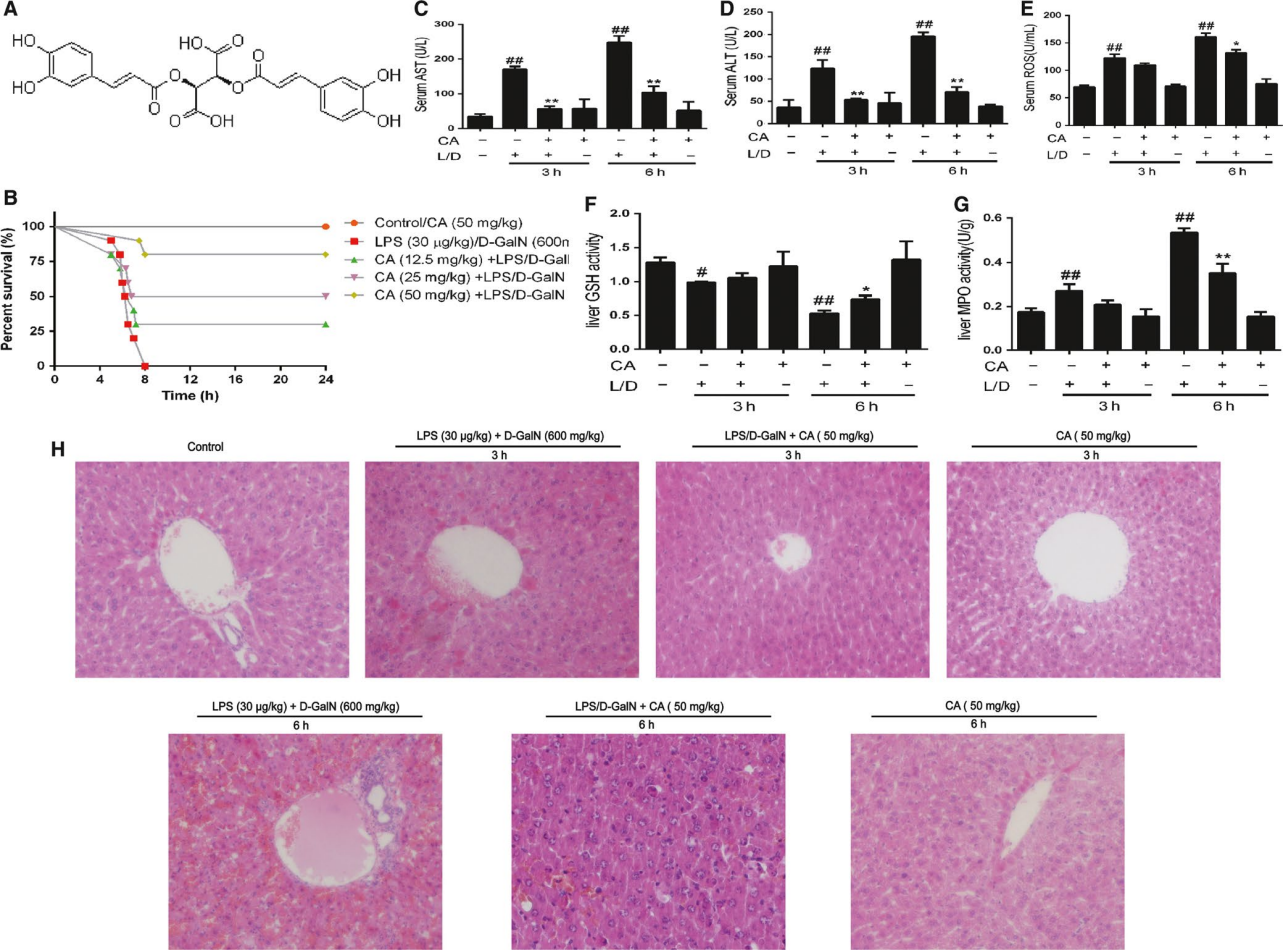
CA decreases the mortality, serum AST, ALT and ROS, MPO in liver, hepatotoxicity and increases the GSH in LPS/d‐GalN‐induced acute liver injury. The mice were injected LPS/d‐GalN to induce acute liver injury, and mice were injected CA (12.5, 25, 50 mg/kg) in the treatment group 1h before the LPS/D‐GalN. And we recorded the mortality in different group in 24 h. Treated by CA (50 mg/kg), the mice were injected LPS/d‐GalN 1 h later. 3 and 6 h later, the liver of mice was fixed to stain with haematoxylin and eosin. A, the constitution of CA. The mortality was shown in B. Sections shown in H in each group were pictured by optical microscope (magnification × 200). Meanwhile, the results of MPO and GSH in liver are pictured in F and G. Besides, we collected the blood of mice to detect the serum AST, ALT and ROS is shown in C, D and E. We took three independent experiments, and the data were shown as means ± SD. ^#^ *P* < .05 vs the control group; ^##^ *P* < .01 vs the control group; *P < .05 vs the LPS/d‐GalN group; **P < .01 vs the LPS/d‐GalN group

Serum ALT, AST and ROS increased with LPS/d‐GalN treatment. 3 hours later, ALT, AST and ROS were slightly increased, while after 6 hours, the level of AST, ALT and ROS was significant increased shown in Figure 1C,D,E. And with the CA treatment, the AST, ALT and ROS were decreased.

### 3.2 CA restrains the level of MPO and increase GSH in mice

MPO, one of the peroxisomes, plays an important role in inflammation. Combined with three amino acids, GSH is an important antidote and antioxidant in organism. As it is shown in Figure 1F and G, CA can restrain the MPO which was increased after treated by LPS/D‐GalN. Meanwhile, treated with CA, level of GSH in liver increased.

### 3.3 CA decreases the mortality of LPS/D‐GalN‐induced ALF

Acute liver failure is a kind of infrequent and lethal disease. After injected CA and LPS/d‐GalN, mice were monitored for 24 hours. The results indicated that mice only treated by CA (50 mg/kg) and the control group were all alive after 24 hours. However, in LPS/d‐GalN group, mice died at 6 hours, and all mice in this group died in 8 hours. Although the mice treated by CA (12.5, 25, 50 mg/kg), the mortality was decreased to 30%, 50% and 80% with dosage dependent (Figure 1B).

### 3.4 CA protects mice form LPS/d‐GalN‐induced ALF in hepatotoxicity

H&E stain sections (5‐μm‐thick) of liver were detected to obtain visual pathological picture. In Figure 1H, the liver of mice had severer intrahepatic haemorrhage and necrosis over time in LPS/d‐GalN group, whereas CA can protect mice form ALF. However, only treated by CA had not found hepatotoxicity compared to control group.

### 3.5 CA activated Nrf2‐HO‐1 pathway in mice

**Figure 2:**
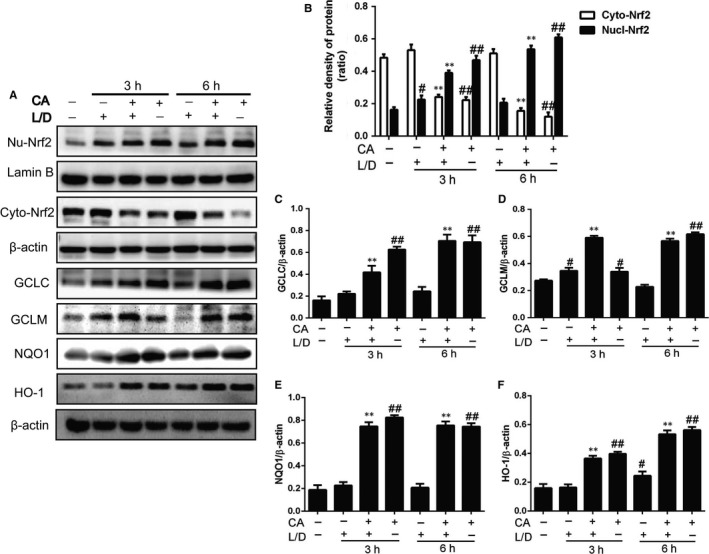
CA activated Nrf2 ‐ HO‐1 pathway in mice. After the treatment of CA, LPS/d‐GalN was given to mice. After 3 and 5 h, we used the liver tissue for Western blot. The results were compared with the internal control (lamin B and β‐actin). The data were collected for data shown as means ± SD of three independent experiments. ^#^ *P* < .05 vs the control group; ^##^ *P* < .01 vs the control group; **P < .01 vs the LPS/d‐GalN group

In this study, we detected the Nrf2 in nucleus and the expression of antioxidase such as GCLC and GCLM. As the results showed in Figure 2, treated by CA, the level of Nrf2 in nucleus was significantly improved with time‐dependent. With the activation of cytoprotective genes, the expressions of HO‐1, NQO1, GCLC and GCLM were improved. Moreover, the results indicate that the Nrf2 in treatment group in cytoplasm was in low level.

### 3.6 CA regulated AMPK pathway in mice

**Figure 3:**
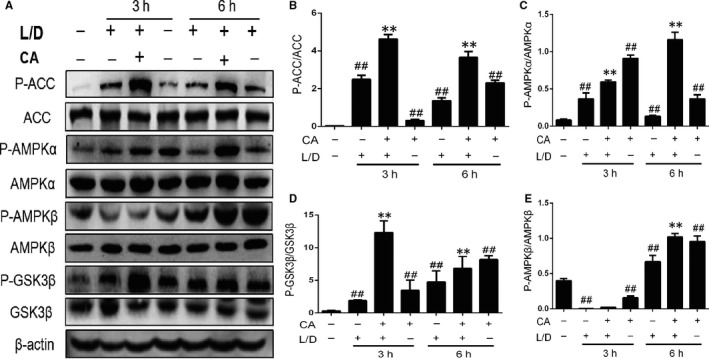
CA regulated the expression of AMPKα, AMPKβ, ACC and GSK3β protein in mice. Before injected LPS/d‐GalN, the mice were injected CA for protection. And we collected the liver (3 and 6 h after the injection of LPS/d‐GalN) to investigate the change of AMPKα, AMPKβ, ACC and GSK3β. We took three experiment independent to obtain results and the data shown as means ± SD. ^##^ *P* < .01 vs the control group; **P < .01 vs the LPS/d‐GalN group

AMPK, a molecular energy sensor, can adjust energy metabolism and physiological processes. The results showed that CA could increase the phosphorylation of AMPKα and AMPKβ (Figure 3) with concentration‐dependent. Acetyl‐CoA carboxylase (ACC), which can catalyse acetyl‐CoA, is a rate‐limiting enzyme. The results showed that phosphorylated ACC increased with CA treatment (Figure 3). Meanwhile, the phosphorylation of GSK3β was up‐regulation after adding the CA.

### 3.7 CA suppress phosphorylation of MAPKs in mice

**Figure 4:**
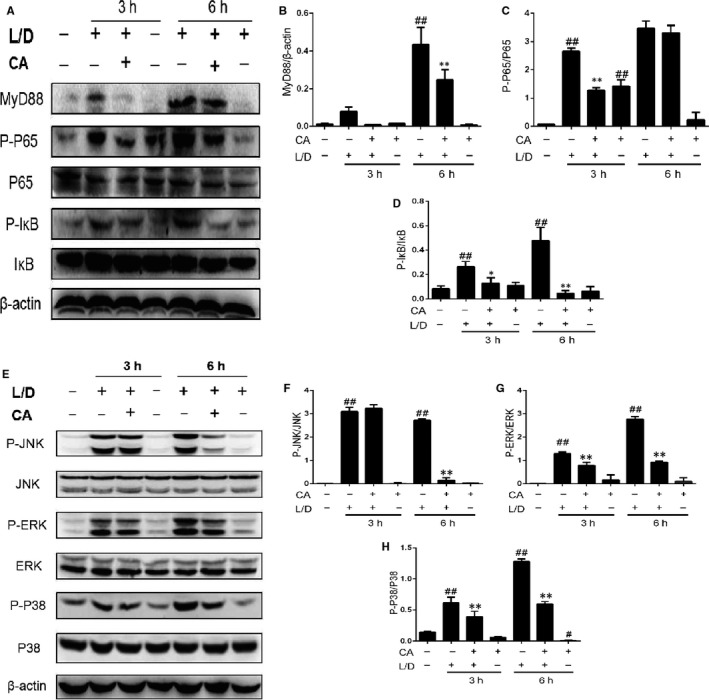
CA suppress phosphorylation of MAPKs and NF‐κB in mice. After the treatment of CA, we took injection of LPS/d‐GalN to mice. And the liver tissue was used to investigate. A‐D, Results of NF‐κB pathway, whereas the MAPKs’ pathway presents in E‐H. Three independent data of experiment were collected, and the data were presented as means ± SD. ^##^ *P* < .01 vs the control group; *P < .05 vs the LPS/d‐GalN group; **P < .01 vs the LPS/d‐GalN group

MAPKs play important roles in growth, differentiation and death of cell. As it is pictured in Figure 4, LPS/d‐GalN induced the excessive P‐JNK, P‐ERK and P‐P38. After treatment of CA, P‐ERK and P‐P38 were obvious decreased at 3 hours. Furthermore, at 6 hours, CA decreased the level of P‐MAPKs.

### 3.8 CA suppress phosphorylation of NF‐κB in mice

Responding activated TLR4 by stimulation of LPS though the Myeloid differentiation primary response gene (MYD88), NF‐κB is a pivotal regulator in inflammation. Figure 4 shows that CA can restrain MYD88, P‐NF‐κB and P‐IκB.

### 3.9 CA regulated NLRP3 pathway in mice

**Figure 5:**
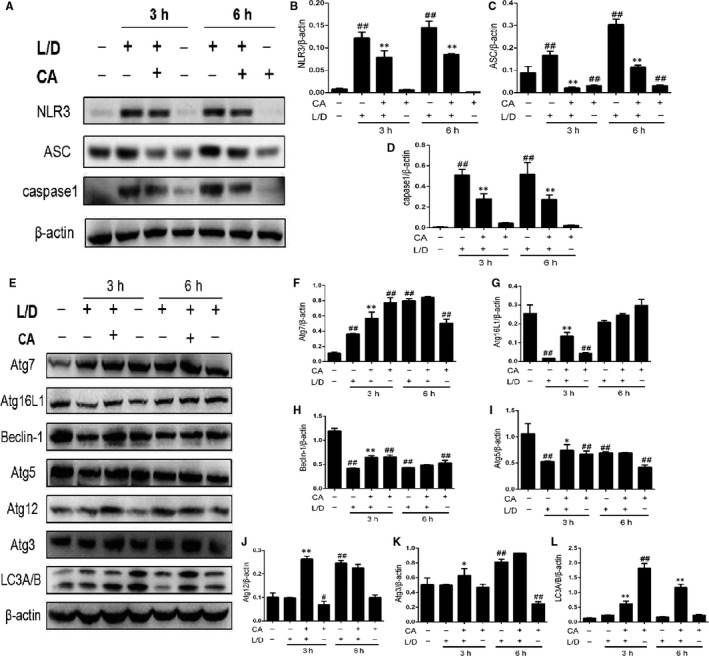
CA regulated NLRP3 and autophagy in mice. Injected with CA, 1h later, we treated mice with LPS/d‐GalN. Western bolt was used to detect the change of protein in liver. A‐D, results of NLRP3 pathway. And the CA can activate autophagy presented in E‐L. Three results from independent experiment were used for statistical analysis with the data shown as means ± SD. ^#^ *P* < .05 vs the control group; ^##^ *P* < .01 vs the control group; *P < .05 vs the LPS/d‐GalN group; **P < .01 vs the LPS/d‐GalN group

NLRP3 can be activated by series of stimulate, such as virus and ATP, and is significant in inflammation. As Figure 5 shows that the CA restrained the ASC and NLRP3, so the caspase1 in treatment groups was lower than LPS/d‐GalN groups.

### 3.10 CA activated autophagy in mice

Autophagy participates in various activities in cells, such as clearance of damaged organelles and causative agent. The results presented in Figure 5 indicate that CA can activate autophagy. The content of Atg16, 12, 7, 5 and 3 Beclin‐1 had varying degrees of increase.

### 3.11 Toxicity of CA on HepG2 cell

**Figure 6:**
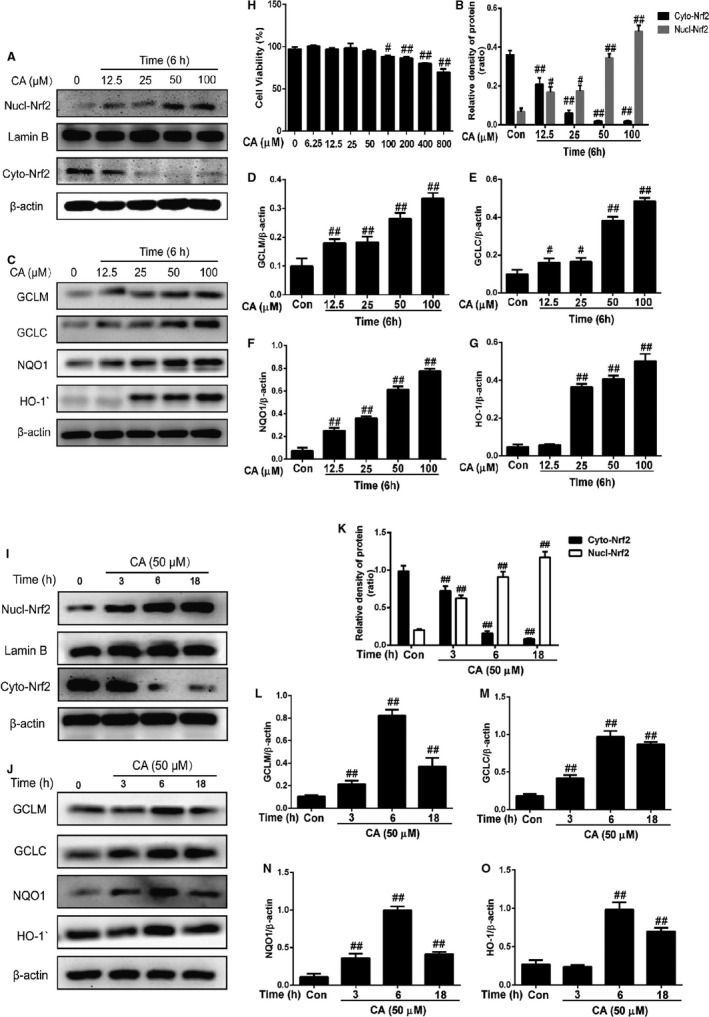
CA (3, 6 or 18 h or 12.5, 25, 50 and 100 μm) regulated Nrf2‐HO‐1 pathway in HepG2 cells and the toxicity of CA on HepG2 cell. We treated HepG2 cells with increasing concentration of CA (12.5, 25, 50 and 100 μm). Six hours later, the cells were collected and lysed by RIPA for Western bolt. HepG2 cells were treated with CA (50 μm) for three time‐points (3, 12 or 18 h). Collected the cells, the total protein was used to detect the expression. The level of Nucl‐Nrf2 was compared with lamin B. And the Cyto‐Nrf2, HO‐1, NQO1, GCLC and GCLM were compared with β‐actin. For the Toxicity of CA on HepG2 cells, we treated the HepG2 cells with CA (0‐800 μm) for 24 and checked the toxicity with MTT. We collected data from three independent experiments, and it was shown as means ± SD. ^#^ *P* < .05 vs the control group; ^##^ *P* < .01 vs the control group

To evaluate the toxicity of CA, we plated HepG2 cell into 96 plates with 5% CO_2_, 37°C. Twenty‐four hours later, we treated the HepG2 by CA (0‐800 μm). The result shows in Figure 6H that CA (0‐50 μm) has no toxicity.

### 3.12 CA regulated Nrf2 ‐ HO‐1 pathway in HepG2 cells (WT and Nrf2^−/−^)

**Figure 7:**
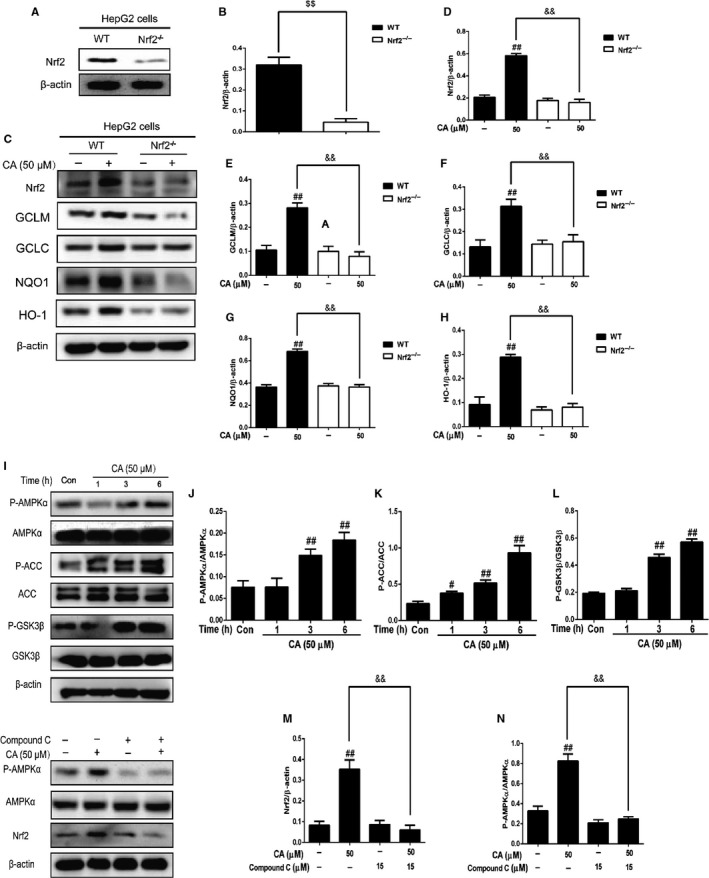
CA regulated the expression of AMPKα, ACC and GSK3β and Nrf2‐HO‐1 pathway in HepG2 cells (WT and Nrf2^−/−^). We treated the HepG2 cells (WT and Nrf2^−/−^) with CA (50 μm) for 6 h. HepG2 cells were treated with CA (50 μm) for (1, 3 or 6 h), and we added compound C with cell treated CA for 6 h. And we investigate the protein with Western bolt. Three independent data were used to statistical analysis. And the results were shown as means ± SD. ^##^ *P* < .01 vs the control group. ^&&^ *P* < .01 vs Nrf2^−/−^ HepG2 cells group

To detect the change of Nrf2‐HO‐1 pathway with CA treatment, we treated the HepG2 cells by CA at three time‐points (3, 6 and 18 hours) or with different concentrations of CA (12.5, 25, 50 and 100 μm 6 hours). Then, protein was collected. As it is shown in Figure 6, CA (50 and 100 μm) accelerated the transfer of Nrf2. Meanwhile, we used 50 μm CA treated HepG2 cells, the results indicated that CA can activated the Nrf2 in 6‐18 hours. Moreover, with the increased of Nrf2, HO‐1, NQO1, GCLC and GCLM were significant increased (Figure 7). Besides, we treated Nrf2^−/−^ HepG2 cells with CA to verify the mechanism. As it has been judged, whether using the CA or not, the expressions of HO‐1, NQO1, GCLC and GCLM were lower compared with those in WT cells.

### 3.13 CA regulated AMPK pathway in HepG2 cells

We have detected the expression of AMPK pathway. After the treatment of CA, the P‐AMPKα was increased (shown in Figure 7). And, phosphoryltion of ACC and GSK3β was improved. Besides, treated by inhibitors of AMPK (compound c), the phosphorylation of AMPKα and the expression of Nrf2 were down‐regulation as it is shown in Figure 7I, M and N.

## 4 DISCUSSION

Acute liver failure can induce multiple organ dysfunction syndrome and has high mortality. Due to the complication of ALF is complex, the existing treatment may also have certain risk. And the ALF is associated with hepatic encephalopathy which can cause serious injury of nerve. LPS/d‐GalN induces ALF which can be used as disease models for drug discovery.^17^ Normally, oxidant is advantageous in signal transduction to make balance, whereas expression of oxidant is out of control leading to process of disease. Extreme oxidative stress and uncontrolled inflammation are crucial in ALF. CA has alleviation in liver injury of mice which have high‐fat diet in previous paper.^18^ Based on existing studies, we investigated effects of CA in ALF induced by LPS/d‐GalN. CA can improve P‐AMPK and Nrf2 in nucleus. Besides, CA can decrease serum AST, ALT and ROS and restrain NF‐κB and MAPK to protect the mice.

The results have indicated that CA decreased serum ALT, AST and ROS. With the deepening of research, the simple increasing of ALT does not represent the injury of liver, whereas the AST/ALT up‐regulation indicates that liver has severe injury. Besides, MPO in liver reduced and the GSH was increased after treatment of CA. Although MPO is activated, it can deteriorate disease without bacteria and has toxicity in normal cell. This makes investigation of restraining overexpression of MPO more important in inflammation. GSH, almost existing in every cell in body, can combine with drugs to withstand the injury of toxicant. And the majority of serum GSH comes from liver to respond break of balance. Meanwhile, the GSH in oxidative stress can stimulate the NF‐κB and MAPK. Furthermore, pathological sections indicated that CA can alleviate ALF, and the mortality was reduced with the CA treatment. Besides, study shows that toxic substance can damage organelle and cellular homoeostasis though the effects on cell signalling cascade pathway.^19^

NF‐κB is combined to IκB in resting time, and it can be activated by proinflammatory factor. CA can restrain P‐IκB and P‐NF‐κB. Present experiments show that NF‐κB can competitively combine with CREB binding protein (CBP) which have influence on the bonding of Nrf2 and CPB.^20^ On the other hand, the increasing HO‐1 can inhibit P‐NF‐κB.^21^ And the H_2_O_2_ can activate NF‐κB pathway. Besides, autophagy is significant in cellular metabolism, and autophagy is associated with various diseases, such as liver injury and cancer.^22^ CA can activate the expression of autophagy gene (such as Atg16 and 3) and Beclin‐1. In previous study, NF‐κB has negative regulation on autophagy though adjusting inducer of autophagy,^1^ whereas autophagy can restrain the activation of NF‐κB though prohibiting the IKK and NIK.^23^ Meanwhile, MAPKs are transporters which can send extracellular signal to intracellular protein in inflammation. The results presented that CA decreased P‐JNK, P‐ERK and P‐P38.

Reactive oxygen species (ROS) and reactive nitrogen species (RNS) are produced in cell metabolism, but excess expression of this substance can induce oxidative stress. And series of reports indicate that ROS is effective modifier of NF‐κB though IKK and other signals. Nrf2 can adjust series of gene transcription to protect oxidative stress and inflammation. Moreover, Nrf2 can restrain the excitation of NLR3 though increasing the expression of NQO1.^24^ It is universal known that the activated Nrf2 can protect the mice form liver injury. Present study shows that various chemical elements from the natural products can protect mice form ALF though Nrf2 pathway.^25^ CA can increase antioxidant enzyme (such as HO‐1 and NQO1) with Nrf2 improved in nucleus in experiment. AMPK is regarded as an energy sensor when it is first discovered. However, with the further research, it is showed that AMPK is significant in energy metabolism and inflammation.^26^, ^27^ For the complicated effects of AMPK, it has been a hotspot for finding the new signal in treatment of diseases. Besides, it has been reported that AMPK can improve the level of Nrf2 in nucleus though restraining GSK3β.^11^ And CA can increase the phosphorylation of ACC and GSK3β, and this may increase the accumulation of Nrf2 in nucleus. We also took study on effect of CA in HepG2 cell. In our investigation, CA can increase NQO1 and HO‐1 by increasing accumulation of Nrf2 in nuclear. Besides, the results demonstrate that CA improves the level of P‐AMPKα and P‐GSK3β. However, when the HepG2 cells were treated by CA with AMPK inhibitors, the level of Nrf2 was obvious decreased. Besides, in Nrf2^−/−^ HepG2 cells treated by CA, levels of GCLC and GCLM were lower compared with HepG2 cells. The results proved that CA can activate Nrf2 pathway which may depend on the AMPK. Besides, the activated AMPK can decrease the level of NF‐κB and TNF‐α.^28^ Meanwhile, P‐AMPK can activate the autophagy by recruiting Atg9 to LC3 autophagosome.^29^

In a word, we mainly proved that CA in activating the AMPK/Nrf2 pathway in ALF in mice while the effects of CA on AMPK/Nrf2 pathway in HepG2 cell were proved. The result indicated that CA can relieve oxidative stress and inflammation, whereas CA can activate the Nrf2 pathway and autophagy. Therefore, our results proved the positive effects of CA in ALF induced by LPS/GalN, which may provide new insights into liver injury treatment.

## CONFLICT OF INTEREST

The authors declare no competing financial interest.

## AUTHORS’ CONTRIBUTIONS

Zheng Li., Guowen Liu and Haihua Feng contributed to the design. Lu Han., Zheng Li, Ye Tian, Lilei Zhao. Lu Ding and Meiyu Jin did the data collection. Zheng Li, Qi Wang, Guown Liu and Haiyan Qin Lu Ding did the analysis. Zheng Li and Lu Han did the writing of the article. Bingyu Shen, Haihua Feng and Guowen Liu did the revisions of the article.

## Data Availability

The data that support the findings of this study are available from the corresponding author upon reasonable request.
